# Schizophrenia and vitamin D related genes could have been subject to latitude-driven adaptation

**DOI:** 10.1186/1471-2148-10-351

**Published:** 2010-11-11

**Authors:** Roberto Amato, Michele Pinelli, Antonella Monticelli, Gennaro Miele, Sergio Cocozza

**Affiliations:** 1 Gruppo Interdipartimentale di Bioinformatica e Biologia Computazionale, Università di Napoli "Federico II" - Università di Salerno, Naples, Italy; 2 Dipartimento di Scienze Fisiche, Università degli Studi di Napoli "Federico II", Naples, Italy; 3 Istituto Nazionale di Fisica Nucleare - Sezione di Napoli, Naples, Italy; 4 Dipartimento di Biologia e Patologia Cellulare e Molecolare "L. Califano", Università degli Studi di Napoli "Federico II", Naples, Italy; 5 Istituto di Endocrinologia ed Oncologia Sperimentale, CNR Napoli, Naples, Italy

## Abstract

**Background:**

Many natural phenomena are directly or indirectly related to latitude. Living at different latitudes, indeed, has its consequences with being exposed to different climates, diets, light/dark cycles, etc. In humans, one of the best known examples of genetic traits following a latitudinal gradient is skin pigmentation. Nevertheless, also several diseases show latitudinal clinals such as hypertension, cancer, dismetabolic conditions, schizophrenia, Parkinson's disease and many more.

**Results:**

We investigated, for the first time on a wide genomic scale, the latitude-driven adaptation phenomena. In particular, we selected a set of genes showing signs of latitude-dependent population differentiation. The biological characterization of these genes showed enrichment for neural-related processes. In light of this, we investigated whether genes associated to neuropsychiatric diseases were enriched by Latitude-Related Genes (LRGs). We found a strong enrichment of LRGs in the set of genes associated to schizophrenia. In an attempt to try to explain this possible link between latitude and schizophrenia, we investigated their associations with vitamin D. We found in a set of vitamin D related genes a significant enrichment of both LRGs and of genes involved in schizophrenia.

**Conclusions:**

Our results suggest a latitude-driven adaptation for both schizophrenia and vitamin D related genes. In addition we confirm, at a molecular level, the link between schizophrenia and vitamin D. Finally, we discuss a model in which schizophrenia is, at least partly, a maladaptive by-product of latitude dependent adaptive changes in vitamin D metabolism.

## Background

During recent decades the study of human evolution has been of increasing interest, due also to the large amount of data now available, concerning populations geographically and widely distributed. Meanwhile, new applications of evolutionary biology to medical problems are being discovered at an accelerating rate [1].

The genetic diversity is one of the most important instruments available to understand our evolutionary history. Differences among individuals belonging to the same population are generally smaller than those of individuals belonging to different populations [2]. This is due to both demographic history and selection that shaped the genome to adapt the genome of different populations to the experienced environment. All loci in a population share the same demographic history, thus they are expected to show similar patterns of variation. But it is a matter of fact that substantial differences in the level of among-population differences across loci exist [3]. It is therefore reasonable to assume that loci showing more among-population differentiation than the rest might identify regions of the genome that have been subjected to selection [4,5].

In 1966 Cavalli-Sforza suggested using measures of population divergence to detect natural selection [6] and Lewontin and Krakauer proposed using for this purpose, Wright's F-statistics [7]. In particular, fixation index (F_ST_) is one of the most widely used descriptive measures of population and evolutionary genetics [8]. F_ST _is directly related to the variance in allele frequency among populations. A small F_ST _value means that the allele frequencies among populations are similar, whereas a large F_ST _value implies that the allele frequencies are different. If natural selection favours one allele among others at a particular locus and only for some particular populations, the corresponding F_ST _for that locus will be larger than in any other locus where genetic drift alone is at work.

Among the environmental factors that strongly influenced our evolutionary history, geographical latitude deserves particular attention. Latitude, indeed, severely affects many natural phenomena such as climate, flora and fauna, light-dark cycle, and all of them, in turn, have an impact on many aspects of our life. For sake of brevity hereafter we refer to all these phenomena simply as "latitude".

Genetic traits following a latitudinal gradient have been observed for several polymorphisms in humans as well as in natural populations of model organisms like *Drosophila *and *Arabidopsis thaliana *[9-12]. The best known example of this kind of spatial variation in *Homo Sapiens *is skin pigmentation. The clinal gradation of skin colouration is correlated with UV radiation levels and represents a compromising solution to the conflicting physiological requirements of photoprotection and vitamin D UV-dependent synthesis [13]. The latter is very important since vitamin D is involved in many health outcomes (e.g. cardiovascular diseases, rickets, pelvic deformities, infections, etc.).

A possible influence of latitude on the circadian phenotype has also been suggested [14,15]. Circadian rhythm is a ubiquitous feature of living systems. Daylight hours vary with latitude and seasons therefore adaptability of circadian clocks is of fundamental importance for the adaptation of organisms to the alternating light/dark cycles.

Another example of spatial variation is human body size and shape. These phenotypes show a correlation with climate (that in turn has a strong relationship with latitude) suggesting also for humans the adaptation to the classical ecological rules that individuals living in colder regions are bulkier and have shorter limb lengths [16,17].

Nevertheless, it is also possible that an allelic variant increasing the fitness of individuals at particular latitudes will no longer be advantageous or even increase the risk for some pathologies at different latitudes. In particular, several diseases show latitudinal clinals. A well known example regards sodium homeostasis. It have been postulated that in hot climate regions, genetic variants inducing enhanced sodium retention were positively selected. This adaptive process would allow a proper vascular tone and salt storage in conditions of excessive sweating. These same variants, when carried by individuals migrated in colder climates (i.e. African American), would increase the risk for sodium retention-related hypertension [18]. Supporting this hypothesis, several studies reported a strong correlation between latitude and the frequencies of hypertension susceptibility variants [18,19].

In addition, there is growing evidence supporting the idea that these diseases are frequently due to a negative by-product of adaptive changes during human evolution [20]. This is the case when natural selection favours a vital phenotype at the price of predisposing to some other pathology that, for example, do not directly affect the reproduction or are characterized by a late onset-age. Indeed, contrasting forces often affect the outcome of natural selection. For instance, depigmentation is crucial for vitamin D synthesis at higher latitudes but it also exposes a higher risk for skin cancer. As most individuals do not develop cancer until they are past their reproductive age, from an evolutionary point of view, skin cancer represents a less powerful selective force than vitamin D availability in serum [21].

Many other common diseases like different types of cancer, dismetabolic conditions, schizophrenia, Parkinson's disease, etc. have an incidence following a latitudinal gradient [22-25]. However, the relative importance of variation of environmental exposures or genetic predisposition is not yet fully defined. In some cases, a genetic adaptation has been suggested [20,22], even if the direct target of this process is often unclear.

In this paper, we investigated the latitude-driven adaptation phenomena, for the first time, on a wide genomic scale. In particular, we selected a set of SNPs and genes showing signs of latitude-dependent population differentiation, and by a biological characterization of the genes, we found enrichment for neural-related processes. In light of this result, we investigated whether genes associated to pathological phenotypes, namely psychiatric and neurological diseases, were enriched for Latitude-Related Genes (LRGs). Remarkably, we found a strong enrichment of LRGs in the set of genes associated with schizophrenia. In an attempt to try to explain this possible link between latitude and schizophrenia, we investigated their association with vitamin D, which had been previously associated, separately, to both of them. Our findings suggest a molecular link among latitude, schizophrenia and vitamin D.

## Results

A set of SNPs showing high levels of latitude-dependent population differentiation was selected by using a two-step approach. Our starting point consists of genotype data concerning about 660,000 SNP loci of 938 unrelated individuals from 51 populations of the Human Genome Diversity Panel [26].

The first step was the estimation of the population differentiation level of each SNP. After the exclusion for minor allele frequency and for SNPs falling in intergenic regions, we obtained a set of 224,501 SNPs. For all of them we calculated the fixation index (F_ST_) according to the Weir and Cockerham estimator [27], and to select SNPs with high levels of population differentiation, we extracted those at the top of the empirical distribution of F_ST _values. Because of the differences existing between the distribution of F_ST _values for autosomic and X-linked SNPs [28], these two sets were handled separately. In particular, we selected 22,132 autosomic and 459 X-linked SNPs falling in the top 10% of their own distributions (namely with F_ST _value greater than 0.153 and 0.262, respectively).

In the second step, we computed for each SNP the absolute value of correlation between the frequency of the ancestral allele in the 51 populations and the absolute value of geographical latitude of the population location. We again handled the distributions of correlation values of autosomic and X-linked SNPs separately because of their differences. Moreover, since population sizes were very different, we computed the correlation by taking into account the number of individuals in each population. We selected the autosomic and X-linked SNPs with absolute value of correlation greater than 0.567 and 0.575 respectively, corresponding to the highest 10% of their respective distributions. We finally obtained two sets of 2193 autosomic and 46 X-linked SNPs corresponding to 1307 and 29 unique genes. Hereafter, we denote by LRGs (Latitude-Related Genes) this set of genes whose SNPs showed both high FST (mean = 0.230) and high latitude correlation (mean = 0.616) values (Additional file 1).

High F_ST _values can be produced both by selection and by demography. The effect of population histories is potentially the major confounding factor in the interpretation of genetic differences among populations. To roughly estimate the extent of phenomena that could be ascribed to natural selection in our LRG set, we used an approach similar to that proposed by Barreiro et al. [29]. We found in LRG an enrichment of non-synonymous SNP of about 30% (p = 0.016, Fisher's exact test; Additional file 2). This finding seems to indicate that, at least in part, LRGs set is enriched for genes under selective pressure.

As a further test for the procedure, we chose a well-known latitude dependent phenomenon such as skin pigmentation and we checked whether LRGs were statistically overrepresented in the set of genes associated to this phenotype. As expected a significant enrichment was found (7 out of 24 genes in common, Fisher's exact test p-value 0.0003).

To characterize the set of LRGs we used two methods, tissue localization and functional characterization. Firstly, we explored the tissue localization of proteins encoded by these genes. To achieve this, we used the Database for Annotation, Visualization and Integrated Discovery (DAVID) focusing on the "Uniprot Tissue" list, a curated list of localization based on literature mining. We found that genes expressed in brain and brain-related tissues were significantly enriched in LRGs, accounting for more than a half of them (Table 1).

**Table 1:** Enrichment in tissue for LRGs computed using the DAVID's Uniprot Tissue category

Tissue	LRGs count (%)	p-value*
Brain	683 (56.6%)	3 × 10^-18^

Amygdale	112 (9.3%)	8 × 10^-6^

Thalamus	76 (6.3%)	1.4 × 10^-4^

For each tissue is reported the number and the percentage of LRGs expressed in the tissue and the significance of that enrichment.

* Fisher's exact test, Bonferroni adjusted

LRGs were also functionally characterized by looking for overrepresentation of Gene Ontology (GO) annotation terms [30]. Because of the high redundancy of GO, we used the Model-based Gene-Set Analysis (MGSA) method included in Ontologizer 2.0 [31,32]. This promising and novel approach analyses all categories together by embedding them in a Bayesian network. Differently from other methods, it provides for each term a marginal posterior probability that reflects a measure of certainty in its involvement in the process. Following the authors' recommendation, we repeated the analysis 20 times in order to see whether the reported marginal probabilities of the top terms fluctuated. We found that two terms out of the three showing a posterior probability above 0.5 consistently among the 20 runs were related to neural processes (Table 2).

**Table 2:** Enrichment for GO terms by LRGs

Name	Sub Ontology*	**Marginal mean (Min-Max)**^**§**^	LRGs count	Total Count
Synapse (GO:0045202)	CC	0.998 (0.980 - 1)	60	351

Neuropetide signalling pathway (GO:0007218)	BP	0.793 (0.764 - 0.828)	13	86

Cell morphogenesis (GO:0000902)	BP	0.734 (0.680 - 0.789)	58	420

For each term is reported the sub ontology to whom it belongs, the mean, minimum and maximum marginal posterior probability of being involved and the number of genes annotated in the list of LRGs and in total.

* CC: cellular component; BP: biological process

^§ ^Among 20 runs

**Figure 1:**
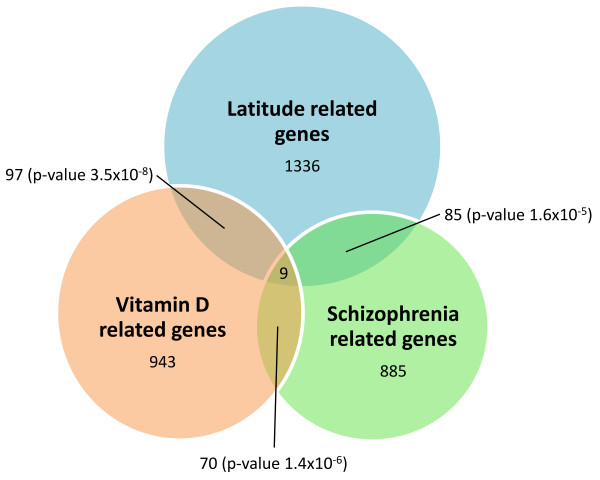
**Overlaps among latitude, vitamin D and schizophrenia related genes**. In each circle is reported the number of genes present in the list. For the two-way intersections is reported the size and the significance of the overlap (Fisher's exact test).

All these results suggested a further investigation about a possible relationship between LRGs and genes involved in neuropsychiatric diseases related with latitude. Indeed, several neurological diseases were previously described to have a latitude-shaped incidence and/or prevalence [23,25,33]. To perform this task we compared the list of LRGs with publicly available collections of genes involved in schizophrenia, multiple sclerosis, Parkinson's and Alzheimer's disease [34-37]. While there is a weak or non significant overlap with genes related to multiple sclerosis, Parkinson's and Alzheimer's disease, we found a significant enrichment of 85 LRGs in genes related to schizophrenia (Fisher's exact test, Bonferroni adjusted p-value 1.6 × 10^-5^; Table 3, Figure 1 and Additional file 3). We found a similar enrichment by comparing the list of LRGs with other lists of schizophrenia related genes (Additional file 2).

**Table 3:** Enrichment of neuropsychiatric diseases lists by LRGs

Disease	Overlap with LRGs	Total count	P-value*	**Adjusted p-value**^**§**^
Schizophrenia	85	885	4 × 10^-6^	1.6 × 10^-5^

Parkinson's disease	40	490	0.021	0.084

Multiple sclerosis	16	178	0.058	0.232

Alzheimer's disease	45	618	0.075	0.3

For each disease genes list, it is reported the number of LRGs present, the total size of the list and the significance of the overlap.

* Fisher's exact test; ^§ ^Bonferroni adjusted p-value

We then investigated for possible latitude-dependent biological mechanisms linking latitude to neural development. An important factor hypothesized to be both latitude dependent and neural development-related is vitamin D. We checked for an enrichment of LRGs in Vitamin D Related (VDR) genes by manually creating a list of 943 genes that broadly comprised the most important processes in which it is involved, since a comprehensive list is not yet present in the literature (Additional file 4). To achieve this, we merged: a list of 6 genes implied in the metabolism of vitamin D obtained from Reactome, a list of 26 genes in the pathway of the control of the expression by vitamin D receptor from Biocarta and a list of 911 genes from a large-scale identification of 1,25(OH)_2_D_3 _target genes by Wang and colleagues [38].

We computed the overlap between LRGs and VDR genes and found a significant overlap of 97 genes (p-value 3.5 × 10^-8^, Fisher's exact test; Figure 1 and Additional file 3). This result suggests that VDR genes show signature of latitude-dependent population differentiation. Also the overlap of 70 genes between VDR and schizophrenia related genes was significant (p-value 1.4 × 10^-6^, Fisher's exact test; Figure 1 and Additional file 3), confirming the role of vitamin D in schizophrenia pathogenesis.

**Figure 2:**
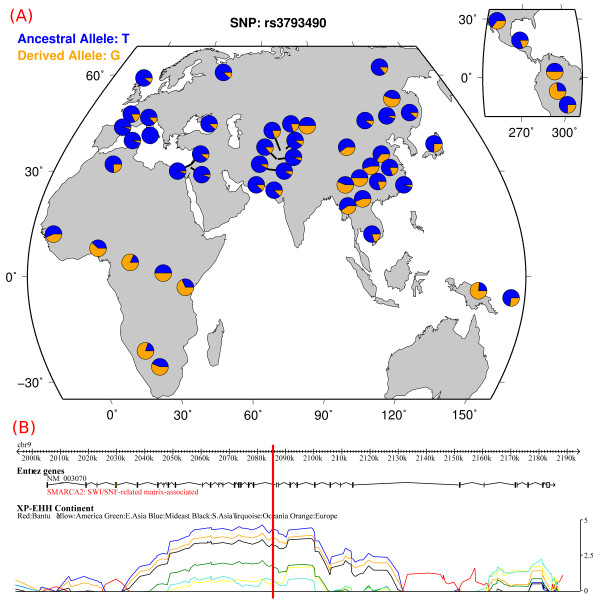
**Alleles geographic distribution (A) and cross-population extended haplotype homozygosity (B) for the SNP rs3793490**. Images were readapted from the "HGDP Selection Browser". The red line marks the SNP's position inside the *SMARCA2 *gene region.

Finally, we found 9 genes (*SMARCA2, MITF*, *DLGAP1*, *MAGI1*, *IL4R*, *NTRK3*, *RUNX1*, *PPP3CA*, and *INPP4B*) in common among those ones related to latitude, vitamin D and schizophrenia (Figure 1). We checked whether or not in these 9 genes belonged SNPs, selected by our procedure, that were previously studied in relationship to either schizophrenia or vitamin D related phenotypes. One SNP resulted from the analysis, rs3793490 (F_ST _= 0.202, correlation = 0.626), an intronic SNP of the *SMARCA2 *gene. In Figure 2 is reported its alleles geographic distribution (A) and the values of cross-Population Extended Haplotype Homozygosity (XP-EHH) (B) of the genomic region. This test detects alleles that have risen to high frequency rapidly, enough that long-range association with nearby polymorphisms (the long-range haplotype) have not been eroded by recombination. The analysis showed a strong sign of recent selective pressure.

## Discussion

Many natural phenomena are directly or indirectly related to latitude. Living at different latitudes has consequences in being generally exposed to different climates, diets, light/dark cycles, etc. Therefore, it is reasonable to presume that exposure of individuals to different latitudes could have shaped genetic background as a result of the adaptation process. Indeed, relationships between allelic frequencies of specific genes and latitude have been identified in plants [39-41] and animals [42,43].

Previous studies in humans, at specific loci, have found evidence of correlation between allelic frequencies and latitude of sampled populations. For example allelic frequencies of a genetic polymorphism in the prion gene *PRNP *showed a clear correlation with the latitude within Europe [44] and one in the *ACP1 *worldwide [45].

To explore possible genetic adaptations to latitude, within this work we defined a set of latitude-related genes (LRGs) following a two step approach. Firstly, we identified SNPs with a high level of population differentiation (F_ST_) with the aim to enrich for variants under selective pressure. From these we then extracted those SNPs showing high values of correlation of allelic frequencies with the geographical latitude (Additional file 1). To the best of our knowledge this is the first search at a wide genomic level for loci showing latitude-dependent populations differentiation.

Both functional characterization and expression localization of LRGs resulted in a strong enrichment of neural-related processes (Tables 1 and 2). The relationship between neural development and latitude is partially known. In particular, there exists evidence of a latitude correlation of some physiological neural-related phenotypes. It was reported for humans that cranial capacity is different between populations, probably, as results of adaptation for brain cooling and that the craniofacial diversity results from the tissues of neural crest origin [17]. It is worth stressing that although population differences are observed in these phenotypes, no relationship exists with mental functioning. Also, for several pathological neural phenotypes there has been described previously a relationship with latitude. For multiple sclerosis and schizophrenia there was a latitudinal variation in incidence and prevalence [25,33]. An association between mortality related to Parkinsonism and birthplace geographical latitude was also found [46].

When we compared the list of LRGs with those containing genes associated to neurological and psychiatric diseases, we found a vanishing enrichment of LRGs in genes related to multiple sclerosis, Alzheimer's and Parkinson's disease (Table 3). This is not surprising. Concerning Alzheimer's and Parkinson's diseases, it should be noted that the relationships with latitude reported in the literature are not largely confirmed as in the case of the other two diseases. Multiple sclerosis has a strong immunity component in the etiology. Since genes involved in immunity related processes usually exhibit low levels of F_ST _[4,28], they are likely to be excluded by our procedure, in this way weakening the enrichment.

In contrast, we found a strong significant enrichment of LRGs in genes associated with schizophrenia (Table 3, Figure 1 and Additional file 3). The correlation of schizophrenia prevalence with latitude is both large and robust [47]. In previous surveys it was noted that there is a strong tendency for schizophrenia prevalence to increase with increasing latitude [25,48]. The present result also agrees with that obtained in one of our previous works, based on different data and different statistical approaches. In this previous study, we explored for signs of natural selection in genes associated to complex diseases, and the strongest level of population differentiation was observed in genes associated to psychiatric diseases [28]. In addition, testing for recent selective sweeps in human populations, Crespi and colleagues found significant evidence for adaptive evolution of several genes underlying schizophrenia [20]. Our results may suggest that a latitude-related adaptation occurred for some schizophrenia associated genes, but to which extent this phenomenon is related to the latitude-shaped prevalence of schizophrenia is of course still an open question.

Searching for a mechanism that could connect latitude and schizophrenia at a molecular level, we reasoned that a well-known phenomenon separately linked to both of them is vitamin D. Indeed, vitamin D is essential for normal growth, calcium absorption and skeletal development. The cutaneous synthesis of vitamin D is a function of skin pigmentation and of the solar zenith angle which, in turn, depends on latitude, season, and time of day [49]. With the same dietary intake, the most important determinant for vitamin D levels is considered to be where individuals live, because of the dependence on geographical location of the availability of UV radiation for vitamin D synthesis. Ancestral populations who migrated out of Africa, moving from south to north were exposed to less incident sunlight. It is commonly accepted that, in these conditions, depigmentation was favoured [50]. This adaptation process was compulsory, since pelvic deformities due to vitamin D deficiency could prevent normal childbirth, but it is reasonable to suggest that also other molecular mechanisms apart from depigmentation could have been subject to selective pressure in order to increase the levels of vitamin D in the body.

To test this link at a genomic level, we created a hand-curated list of genes that, at different levels, are related to vitamin D (see Results; Additional file 4). We found among vitamin D related genes a significant enrichment for genes latitude-related (Figure 1 and Additional file 3). This result may suggest that vitamin D related genes could have been subject, at least in part, to a latitude-dependent adaptation, supporting the hypothesis that selection also acted on other mechanisms different from depigmentation.

On the other hand, the relationship between vitamin D and schizophrenia is well established. Vitamin D receptors were found in most tissues other than those classically involved in the vitamin D action (bone, gut, kidney, etc.). In particular, receptors for vitamin D are widely distributed in the nervous system and vitamin D has been recently implicated in brain function [51]. There is growing evidence that low vitamin D levels adversely impact on brain development [52]. In mice, it has been suggested that changes in brain development induced by prenatal vitamin D deficiency lead to specific functional alterations in hippocampal synaptic plasticity [53]. Also in humans, maternal vitamin D insufficiency has been associated with childhood rickets and longer term problems including schizophrenia [54]. In addition, epidemiological data suggest that vitamin D deficiency may be associated with increased risks of mental health disorders such as schizophrenia [55]. Despite the consistence of this relationship, to our knowledge studies connecting schizophrenia and vitamin D at a molecular level are not yet available.

We found a significant overlap of 70 genes between schizophrenia and vitamin D related genes (Figure 1 and Additional file 3). Our analysis provides the first hint, at a genomic level, of the existence of a relationship between them. The largest part of vitamin D related genes in our list is made by genes differentially expressed in epithelial cells after treatment with the biological active form of vitamin D [56]. It is therefore possible that the same genes could be regulated by vitamin D levels also in neurons during brain development that, in turn, have been associated to schizophrenia [47].

According to our hypothesis that vitamin D could be the link between latitude and schizophrenia, we focused on the 9 LRGs present in both the lists of vitamin D and schizophrenia related genes (Figure 1). Among these genes, that were related to schizophrenic phenotypes, several of them were also previously described in association with bone development. However, no evidence of selective adaptation was present in the literature until now.

For example, one of these genes is the neurotrophic tyrosine kinase receptor type 3 (*NTRK3*) which encodes a member of the NTRK family. These neurotrophins (NTs) receptors are best known for their role in the differentiation and survival of various types of neurons [57]. Gene expression of *NTRK3 *has been reported to be reduced in patients with schizophrenia [58,59]. Furthermore, it has been suggested that the *NTRK3 *gene influences hippocampal function and may modify the risk of schizophrenia [60]. Nevertheless, NTs and their receptors are also produced by a growing list of non-neuronal cells [61] including osteoblastic cell lines [62]. NTs receptors were observed in the bone forming area during fracture healing. *NTRK3 *was observed in osteoblast like cells and hypertrophic chondrocytes [63].

Another example is *PPP3CA *(protein phosphatase 3 catalytic subunit alpha isoform). This gene, also known as calcineurin A alpha, acts as a calcium-sensor and regulator of calcium homeostasis. For this reason, it shapes calcium and cyclic AMP dependent processes like synaptic activity, receptor desensitization, cell survival and neuroplasticity. Expression of *PPP3CA*, previously described by microarrays analysis from cortical [64] and rat hippocampus tissues [65], was found down regulated in schizophrenic anterior temporal lobe [66]. On the other hand, *PPP3CA *is also expressed in osteoclasts, playing a role in the regulation of bone resorption and its deletion results in osteoporosis [67,68].

At least in one case, we also found independent evidence of an adaptive process, reasonably suggesting a molecular mechanism. This is the case of the *SMARCA2 *(SWI/SNF related, matrix associated, actin dependent regulator of chromatin, subfamily a, member 2) gene. The protein encoded by *SMARCA2 *(also known as *BRM*) is part of the large ATP-dependent chromatin remodelling complex SNF/SWI, which is required for transcriptional activation of genes normally repressed by chromatin. Mammalian SWI/SNF complex actually consists of a small series of compositionally distinct assemblies distinguished by the presence of alternative subunits. The complexes contain either one of two closely related alternative ATPases: Brahma (*BRM *i.e. *SMARCA2*) or Brahma-related gene 1 (*BRG1*). The combinatorial assembly of these complexes could account for the specificity of their functions in different tissues and development phases. SWI/SNF components and DNA replication-related factors form, in turn, a human multiprotein complex (WINAC) that directly interacts with vitamin D receptor [69]. WINAC and vitamin D receptor are targeted to vitamin D responsive promoters in the absence of ligand to both positively and negatively regulated genes. WINAC may rearrange the nucleosome array around the positive and negative vitamin D responsive elements (VDREs), thereby facilitating the coregulatory complexes access for further transcription control. Subsequent binding of coregulators requires ligand binding. Several studies have revealed that one family of the SWI/SNF complexes based on the *BRG1 *and *BRM *ATPases has particular critical dosage-dependent roles in the development of the nervous system [70,71].

One of the five SNPs with the highest levels of both population differentiation and correlation with latitude that we identified at this locus was rs3793490. In a previous study this SNP was associated with *SMARCA2 *expression levels in the human brain [72]. In particular, the T variant, whose frequency increases in a latitude dependent way, is associated with low *SMARCA2 *expression levels. Much evidence suggests that low levels of *SMARCA2 *may play a role in the pathophysiology of schizophrenia. *SMARCA2 *knockout mice showed impaired social interaction and prepulse inhibition. In the mouse brain, psychotogenic drugs lowered *SMARCA2 *expression while antipsychotic drugs increased it [72]. On the other hand, in MC3T3-E1 cell line, deficiency of *SMARCA2 *results in an accelerated rate of mineralization with higher levels of expression of osteogenic markers [73]. In addition, the most prominent phenotype of *SMARCA2 *null mice is a larger (about 14% more than normal) body size with a disproportionately increased bone and muscle mass [74]. Putting together all this data seems to suggest that SMARCA2 deficiency is associated with different effects on bones and neurons. It is possible to speculate that a selective pressure could have favoured a haplotype containing the T allele in an environment with low vitamin D availability, for its positive effect on the bone phenotype. In turn, the low *SMARCA2 *expression levels linked to this variant could be responsible for the increased risk of schizophrenia. The XP-EHH analysis that we performed (Figure 2) confirms the presence of a strong recent positive selection in the *SMARCA2 *locus. Interestingly, the homozygosity geographical pattern detected by XP-EHH recognises the effect that we found with correlation analysis. These results agree with a previously described hypothesis of schizophrenia being, at least partly, a maladaptive by-product of adaptive changes during human evolution [20].

In Figure 1, besides the described overlaps among the lists used, large non-intersecting areas exist. This can be partially due to technical limitations of our work. Anyway, these areas of non-intersection were, at least in part, expected. As stated, latitude acts directly or indirectly, on a wide variety of phenomena, therefore it is expected that its effects are not limited just to vitamin D and neural development. For similar reasons, we expected that also the link between latitude and schizophrenia cannot be completely explained by vitamin D. For example, in the intersection between LRGs and genes related with schizophrenia (Additional file 3) there is a gene involved in the circadian rhythms, *TIMELESS*.

Circadian rhythm consists of light and dark phases which coincide with the phases of the solar day and that is, obviously, correlated with the different photoperiods existing at different latitudes. *TIMELESS *is required for normal progression of S-phase and is involved in the circadian rhythm autoregulatory loop. Associations between circadian gene polymorphisms, including *TIMELESS*, and some mental disorders have been found, including schizophrenia [75]. In addition, expression of *TIMELESS *was investigated in the pitcher-plant mosquito, *Wyeomyia smithii*, and was found to vary with latitude of origin. This suggests that other mechanisms should be taken into account [76].

It is worth stressing that this work represents just an initial exploration of this complex problem and can suffer from some limitations. The first aspect to take into account is the arbitrary choice of the thresholds used as inclusion criteria in the list of LRGs. However, it should be underlined that our aim was just to obtain a list enriched for genes showing both high levels of population differentiation and correlation with latitude. Another important aspect is that the largest part of our analysis is based on hand-curated lists. Nevertheless, it should be noted that these lists are widely used [77,78]. The only exception is the list of vitamin D related genes that we had to build *ex novo *since no others were present in literature. In addition, a cause-effect relationship can never be conclusively established based only on a reciprocal enrichment between sets of genes. Moreover, alternative explanations of the observed enrichments can be imagined. For example, we cannot exclude the presence of an unknown common factor, different from vitamin D, linking together schizophrenia and latitude. A further limit of our approach concerns the gene level analysis. In fact, focusing on genes rather than on SNPs, does not allow to conclusively assess whether variants related to latitude coincide with those involved in the studied phenotypes. In other words, even if a gene is associated to both latitude adaptation and schizophrenia, we cannot exclude that this is due to functionally independent variants. For this reason our conclusions should be corroborated by further and more detailed studies. Finally, we are aware that population differentiation is influenced by demographic history and thus it cannot be straightforwardly interpreted as a sign of natural selection. Some approaches have been proposed to evaluate the impact of natural selection on population differentiation. Under the assumption of neutrality, any set of SNPs, even if classified according to their physical location and functional impact, should show the same degree of population differentiation. According to Barreiro and colleagues, any deviation from this expectation should be attributable to selection [29]. In particular, the authors observed that variants leading to amino-acid changes (non-synonymous mutations) are overrepresented among SNPs showing high level of F_ST_. They interpreted this excess as the result from the action of natural selection. We used a similar approach to analyse our data. We found an excess of non-synonymous polymorphisms in LRGs. This result seems to suggest the appreciable presence of genes under selective pressure in LRGs.

During the preparation of the manuscript, an international team of researchers presented the first detailed analysis of the draft sequence of the Neanderthals' genome [79]. In particular, they showed the presence of interbreeding between Neanderthals and *Homo Sapiens *occurred after the Out-of-Africa migration. Affecting mainly the non-African populations, this genetic flow could mimic a latitudinal effect on the Africa-Europe axis and therefore potentially influences our conclusions. First of all, LRGs are selected according to a worldwide latitude correlation, which is only partially due to the Africa-Europe axis. This is the case, for example, of the rs3793490 SNP (Figure 2) where it is also clear a latitudinal effect out of this axis. In addition, Europeans and Asians share only 1% to 4% of their nuclear DNA with Neanderthals suggesting a limited impact, if any, on our conclusions. Finally, we observed that approximately 10% of LRGs are present in the list of genes that Green et al. showed to have evolved recently in our lineage after we split from Neanderthals, and thus, at least in these cases, we can confidently exclude the influence of interbreeding.

## Conclusions

We found that frequencies of genetic variants involved in brain development, schizophrenia and vitamin D-related processes significantly vary with latitude. We also found a molecular relationship of schizophrenia with both latitude and vitamin D. At least in one case, our results suggest that schizophrenia could be a maladaptive by-product of latitude dependent adaptive changes in vitamin D metabolism. The great importance of latitude in human evolutionary history suggests that also other pathologies should be explored.

## Methods

### Data

The whole analysis is based on genotypes data of 660,918 single-nucleotide polymorphisms (SNPs) in samples from the Human Genome Diversity Panel (HGDP-CEPH), which represents 938 unrelated individuals from 51 populations from sub-Saharan Africa, North Africa, Europe, the Middle East, South/Central Asia, East Asia, Oceania, and the Americas. We removed the two small heterogeneous Southern Bantu populations as in [80]. Genotypic data was retrieved from http://hagsc.org/hgdp/[81] while geographical information was obtained from http://www.cephb.fr/en/hgdp/.

For all the SNPs, we computed the allelic frequencies within each population by using the R package "genetics" version 1.3.4. Additional SNP information about physical positions and SNP-gene mapping were obtained from dbSNP build 129 http://www.ncbi.nlm.nih.gov/projects/SNP. Data from the HGDP-CEPH project and dbSNP were merged in a local MySQL database.

After excluding SNPs with minor allele frequencies less than 5% in all of the populations, we obtained a set of 655,810 SNPs. Since we were interested in performing this study at a gene level, we retained only intragenic SNPs obtaining a final set of 224,501 SNPs. In particular, we considered all SNPs within 2 kb of a gene, according to dbSNP classification. We also excluded the Y-linked SNPs because of scarce numbers.

Genes underlying skin pigmentation were obtained from a study by Myles and colleagues [82]. Genes related to neuropsychiatric diseases were obtained from a set of publicly available comprehensive, uniform and regularly updated database of genes considered involved in schizophrenia, multiple sclerosis, Parkinson's and Alzheimer's disease [34-37]. These databases collect genes related to these phenotypes resulting from different approaches (association, genome-wide, candidate, etc.). We used the January 30^th ^2010 update of these databases, containing 892, 197, 511 and 622 genes, respectively. The list of vitamin D related genes was manually created by merging three different lists of genes related in different ways to vitamin D (Additional file 4). The first list is the "Vitamin D (calciferol) metabolism" pathway by Reactome (REACT_13523.2). The second one was extracted from the Biocarta's pathway "Control of the expression by vitamin D receptor" (h_vdrPathway) as present on the Cancer Genome Anatomy Project http://cgap.nci.nih.gov/. Finally, candidate transcriptional target genes of vitamin D were obtained from a set of genes differentially expressed in SCC25 cells treated with 1,25(OH)_2_D_3 _by a genome-wide microarray analysis [56].

A common problem in using lists of genes is that different types of gene identifiers are used. The effect is that usually only part of the genes is recognized. Therefore, each tool that we used lost a variable number of genes during the analyses. Nevertheless, to obtain the best performances from each tool we decided to provide them with the whole lists. In particular, DAVID recognized 1207 identifiers and MGSA recognized 1101 ids. In addition, overlaps between lists of genes were computed by using MatchMiner web tool, which also takes into account gene aliases and/or different types of gene identifiers [83]. This tool recognized 885 schizophrenia, 178 multiple sclerosis, 490 Parkinson's disease, 618 Alzheimer's disease and 1254 latitude related genes.

### Statistical analysis

For all SNPs we calculated the fixation index (F_ST_) according to the Weir and Cockerham estimator [27] using a previously developed Perl script [28]. We then computed the absolute value of the correlation between the frequency in each population of the ancestral allele and the absolute value of the latitude of the population weighted by its sample size. This was to avoid the overweighting of allelic frequencies inside small populations and was implemented simply by using the weighted mean, standard deviation and covariance.

Statistical significance of overlaps was estimated by using Fisher's exact test, considering 21463 genes (number of distinct gene symbols present in the "refGene" track of UCSC Genome Browser) as background population. All statistical analyses were carried out with R ver. 2.10.1 [84]. The whole study was conducted considering a p-value of 0.001 as statistical significance threshold.

To check for a potential bias toward larger genes (since they can contain more genotyped SNPs), we applied the non -parametric Mann-Whitney test to compare the difference among the lengths of LRGs with respect to the remaining genes in the "refGene" track of UCSC Genome Browser. There is no statistical evidence for a difference (p = 0.07), with median length of LRGs slightly smaller than median of the others.

### Biological characterization

The analysis of gene expression localization was performed by using the Database for Annotation, Visualization and Integrated Discovery (DAVID) v6.7 and the "UP_TISSUE" category [85,86]. The "Uniprot tissue" (UP_TISSUE) list is based on literature mining and reports for each gene in which tissues it has been found to be expressed, by using a curated vocabulary.

Overrepresentation of GO terms was assessed by using Ontologizer 2.0 and the Model-based Gene Set Analysis (MGSA) method. The idea behind this approach is to estimate the marginal posterior probability of a term being enriched by using a Bayesian Network. The greater probability for a GO term being near to 1, the higher is the certainty of its involvement in the process. All parameters were left to "auto", allowing the system to automatically estimate the optimal a priori probabilities and the false positive and false negative rates. Probabilities are estimated by MGSA using 10^7 ^Markov-Chain Monte Carlo (MCMC) steps. Since MCMC is not guaranteed to converge in any a priori defined number of steps, we repeated the analysis 20 times and retained only terms having a marginal posterior probability greater than 0.5 in every run, as recommended by authors.

## Authors' contributions

RA, MP, GM and SC participated in the design of the study and performed the statistical analysis. AM, GM and SC conceived of the study, and participated in its design and coordination and helped to draft the manuscript. All authors read and approved the final manuscript.

## Supplementary Material

Additional file 1 **LRGs**. Excel spreadsheet containing the list of Latitude Related Genes with F_ST _and correlation value for each SNP. Click here for file

Additional file 2 **supp_data**. Additional information about the enrichment of SNP classes and the enrichment of LRGs in various lists of schizophrenia related genes. Click here for file

Additional file 3 **overlaps**. Excel spreadsheet containing the list of genes in each two-ways intersection. Click here for file

Additional file 4 **VDRGs**. Excel spreadsheet containing the list of vitamin D related genes. Click here for file
