# Supplementary material for: Schizophrenia and vitamin D related genes could have been subject to latitude-driven adaptation
Source: BMC Evol Biol. 2010 Nov 11;10:351. doi: 10.1186/1471-2148-10-351 (PMC2996405; doi:10.1186/1471-2148-10-351)
Supplement: Additional file 2 — supp_data. Additional information about the enrichment of SNP classes and the enrichment of LRGs in various lists of schizophrenia related genes. [file 1471-2148-10-351-S2.PDF]

Schizophrenia and Vitamin D Related Genes Could Have Been Subject to Latitude-driven Adaptation

Amato R, Pinelli M, Monticelli A, Miele G and Cocozza S

- SUPPLEMENTARY DATA -

SNP classes enrichment

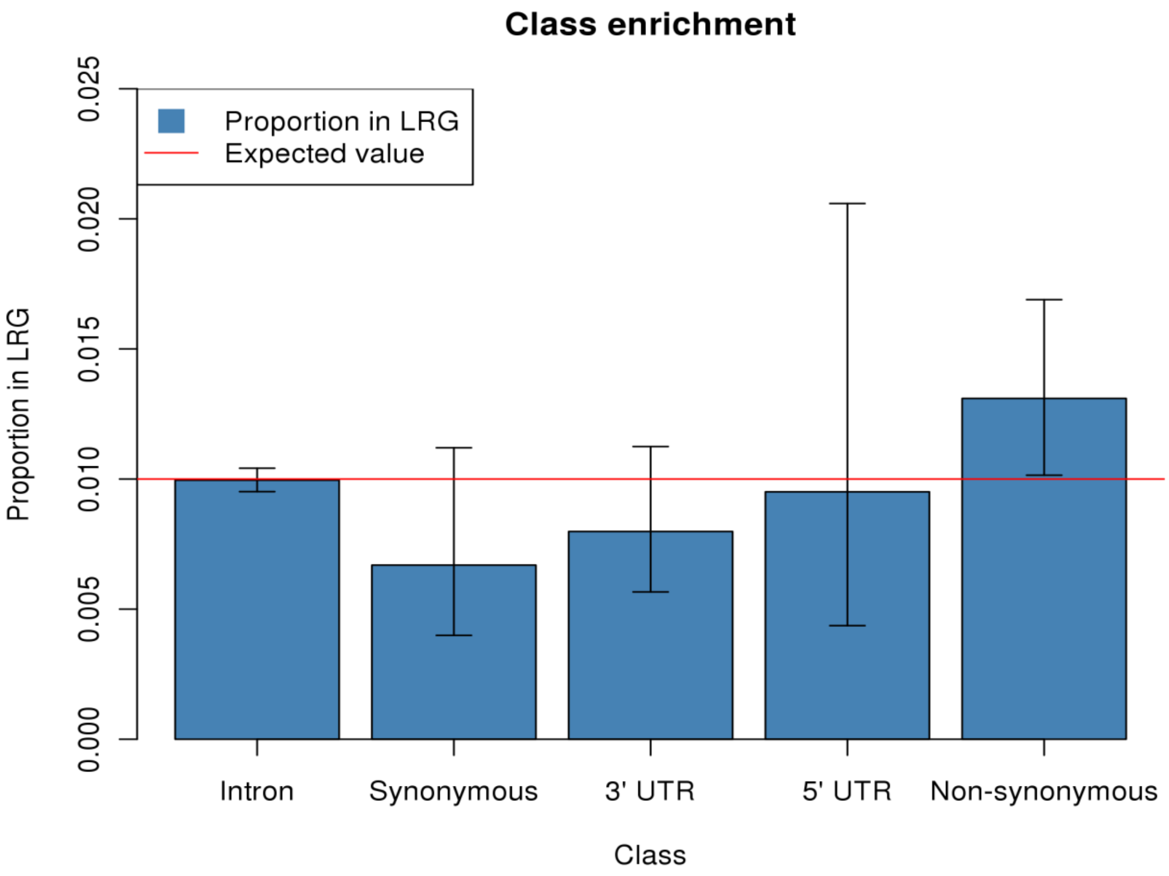

We tried to estimate the extent of population differentiation that could be ascribed to selection following the approach proposed by Barreiro et al (Nat Genet. 2008 Mar;40(3): 340-5). To achieve this goal, in their paper Barreiro and colleagues used a genome-wide approach analysing both genic and non-genic SNPs. The main difference between ours and Barreiro's approach is that, in our study, we considered intragenic SNPs only since we were focused on functional analysis at a gene level. In the above figure we plot the proportion in the set of LRGs of SNPs according to their classes (dbSNP classification). The intronic set does not show any significant variation with respect to the expected 1% (red line). This value represents the expected ratio between the number of LRGs SNPs and the number of all the intragenic SNPs (10% of 10%), under neutral hypothesis. Conversely, non-synonymous SNP class shows an enrichment of about 30% (p = 0.016; Fisher's exact test). This kind of enrichment has been interpreted by Barreiro and colleagues as a signature of selection. Error bars represent the 95% confidence level of the observed proportion. Such

intervals have been obtained in the analytical way by using the Wilson score interval method (Wilson, E. B. J. Am. Stat. Assoc. 1927. 22:209–212).

Enrichment of LRGs in various lists of schizophrenia related genes

We explored if the enrichment found in the used list was also present in pruned sub-lists. For this reason, four different lists were used and all fairly support the presence of a relationship between latitude and schizophrenia:

- **"Association"**
  (http://bioinfo.mc.vanderbilt.edu/SZGR/html/associationdoc.jsp)
  It was obtained from the SchiZophreniaGene (SZGene) database, cleaning data according to a risk-allele evaluation pipeline developed by Sun et al. (Am J Med Genet B Neuropsychiatr Genet. 2008. Oct 5;147B(7): 1173-81). According to this meta-analysis, 278 protein-coding genes were selected having significant p-values using a combined OR method or at least one positive association result in publication. In this gene set we found an enrichment of 27 LRGs (p = 0.0068).
- **"Core"**
  (http://bioinfo.mc.vanderbilt.edu/SZGR/showGeneset.do?gsname=Core)
  It contains genes that have been manually collected to include those that have been commonly considered as candidate genes in expert review or had significant results in the meta-analysis of association studies. Ross et al. (Neuron. 2008. 52(1): 139-53) reviewed the evidence in four domains (association with schizophrenia, linkage to gene locus, biological plausibility, and altered expression in schizophrenia) and suggested 19 genes being candidates. They also included 27 genes with significant meta-analysis results performed by the SchizophreniaGene team. The genes were selected by having a nominally significant summary OR in all ethnic groups or Caucasian samples. After removing redundancy, the core gene set contains 38 genes. In this set we found an enrichment of 7 LRGs (p = 0.0059).
- **"75 genes by COR"**
  (http://bioinfo.mc.vanderbilt.edu/SZGR/showGeneset.do?gsname=GR_COR)
  This list contains 75 genes that were prioritized by ranking about 500 genes from more than 2000 association studies (Sun et al. Am J Med Genet B Neuropsychiatr Genet. 2008. Oct 5;147B(7): 1173-81). This list shows an enrichment of 11 LRGs (p = 0.0041).
- **"173 by Ng et al."**
  (http://bioinfo.mc.vanderbilt.edu/SZGR/showGeneset.do?gsname=GR_Ng)
  This list contains 173 genes and it is based on genetic studies for schizophrenia from four major categories: association studies, linkage analyses, gene expression, and literature search. Genes in these data sets are initially scored by category-specific scoring methods. Then, an optimal weight matrix is searched by a two-step procedure (core genes and unbiased P values in independent genome-wide association studies). Finally, genes are prioritized by their combined scores using the optimal weight matrix. This set shows an enrichment of 19 LRGs (p = 0.0061).
